# Antioxidant (Tocopherol and Canolol) Content in Rapeseed Oil Obtained from Roasted Yellow-Seeded *Brassica napus*

**DOI:** 10.1007/s11746-016-2921-7

**Published:** 2016-11-24

**Authors:** Aleksander Siger, Marzena Gawrysiak-Witulska, Iwona Bartkowiak-Broda

**Affiliations:** 1 Department of Biochemistry and Food Analysis, Poznań University of Life Sciences, Wojska Polskiego 28, 60-637 Poznań, Poland; 2 Institute of Food Technology, Poznań University of Life Sciences, Wojska Polskiego 28, 60-637 Poznań, Poland; 3 Plant Breeding and Acclimatization Institute, National Research Institute, Strzeszyńska 36, 60-479 Poznań, Poland

**Keywords:** Cold-pressed oil, Yellow-seeded rapeseed oil, Peroxide value, Acid value, Canolol, Tocopherols

## Abstract

In this study, the effect of temperature (140, 160, 180 °C) and roasting time (5, 10, 15 min) on the bioactive compound content (canolol, tocopherol and plastochromanol-8) of cold-pressed oil from yellow-seeded rapeseed lines of different colors was investigated. Roasting increased the peroxide value in the seed oils compared to the oils from the control samples. However, roasting did not affect the acid values of the oils, which were 1.15–1.47 and 1.30–1.40 mg KOH/g, for line PN1 03/1i/14 (yellow seeds) and line PN1 563/1i/14 (brown seeds), respectively. In this study, the seeds of line PN1 03/1i/14 were characterized by different changes in canolol content during roasting than the seeds of PN1 563/1i/14. There was a 90-fold increase in canolol for the line PN1 03/1i/14 (768.26 µg/g) and a 46-fold increase for the line PN1 563/1i/14 (576.43 µg/g). Changes in tocopherol and PC-8 contents were also observed. There was an increase in the contents of γ-T and PC-8 in the oils obtained from the seeds roasted at 180 °C for 10 and 15 min. γ-T content increased by 17–18% after 15 min of roasting, whereas the PC-8 content increased twofold.

## Introduction

Rapeseed oil is the third most commonly produced vegetable oil in the world, just behind palm and soy oil. [1]. Black rapeseed is usually grown and there is ongoing work to improve it. To this day, its yield has been improved and double low cultivars have been developed and introduced into production. These show decreases in the amounts of erucic acid (<2%) and antinutritive compounds, with glucosinolates being decreased as well. Strains of rapeseed with modified fatty acid compositions have been developed to expand the uses of rapeseed oil. Currently, work is being done on increasing the amount of active biological compounds such as sterols and tocopherols [2–8]. Studies of yellow-seeded rapeseed are being conducted with the aim of decreasing the dietary fiber contents of the seeds while increasing their oil and protein contents [9, 10].

In recent years, consumer interest in natural, ecological, and low-processed products is on the increase (so-called “green processing”). Cold-pressed oils are considered ecologically friendly and natural by consumers [11]. These oils are not refined, they contain a large number of compounds coextracted with the lipids. Rapeseed oil is characterized by the ideal polyunsaturated fatty acid content with a 2:1 ratio of linoleic (n-6) *versus* linolenic (n-3) fatty acid and high contents of active biological compounds, such as tocopherols, plastochromanol-8, phytosterols and phenol compounds [12–15]. Rapeseed has the highest phenolic content among to other oilseeds (soybean, sunflower), about tenfold, however, phenols remain in the meal after pressing [16]. Phenol compounds, including sinapinic acid and other derivatives, show high antioxidant activity under *in vitro* conditions [17].The main phenol compound of rapeseed is sinapinic acid, which makes up 70% of the total content of free phenolic acids and their derivatives such as sinapine [18]. Other derivatives of sinapinic acid that have been identified include 1-O-β-d-glucopyranosyl sinapate; 1,6-di-*O*-sinapoylglucose; 1,2,6′-tri-*O*-sinapoylgentiobiose; sinapic acid methyl ester, 3-dihexoside-7-sinapoyl-hexoside kaempferol and 3-hexoside-7-sinapoyl hexoside kaempferol [19–23]. During rapeseed roasting and pressing, a sinapinic acid decarboxylation product is formed and transferred to the oil producing an oil enriched with high antioxidant activity compounds [24, 25]. In the literature, data are available on the effect of roasting and microwaving of rapeseed and mustard seeds on the content of native bioactive compounds including canolol [26–30].

However, these studies are concerned with black rapeseed. There is no data on the effect of the roasting process on yellow-seeded rapeseeds, which are characterized by thinner seed coats and lower fiber contents. The goal of this project was to assess the effect of roasting (various times and temperatures) on the contents of canolol, tocopherol, plastochromanol-8 in oils obtained from yellow-seeded rapeseeds. The effect of yellow-seeded rapeseed on the rate of decarboxylation of sinapinic acid was studied.

## Materials and Methods

### Chemicals

All tocopherol homologues (purity > 95% by HPLC) were obtained from Calbiochem-Merck Biosciences (Darmstadt, Germany). *n*-Hexane (HPLC-grade), and 1.4-dioxane (HPLC-grade) were purchased from Merck (Darmstadt, Germany). All other solvents and chemicals used in this study were of analytical grade.

## Materials

Two yellow-seeded winter rapeseed lines were obtained from the Laboratory of Genetics and Quality Breeding of the IHAR-PIB Research Division in Poznań. The origin of the yellow-seeded lines lies in crosses of a spontaneous winter rapeseed mutant with lighter-colored seeds with spotted seed coats—a spring line obtained from earlier *Brassica napus* × *Brassica rapa* crosses [31]. Seed color was determined using a ColorFlex spectrophotometer on a scale from 0 (black) to 5 (yellow) [32].The lines used in the experiments were characterized by different seed colors: line PN1 03/1i/14 was yellow (4.6) and line PN1 563/1i/14 was brown (3.5). This study examined two new breeding lines of yellow rapeseed, differing by seed-coat color.

### Seed Roasting and Cold-pressed Oil Extraction

The control consisted of oil from seeds that had not undergone roasted prior to pressing. The processes of roasting and cold pressing have been presented in an earlier study applied to black-seeded rapeseeds [30]. For each sample, 1000 g of whole rapeseeds were placed in Petri dishes (25 cm in diameter). The seeds were roasted in a UFE55 Universal Oven with forced ventilation (Memmert KG, Schwabach, Germany). Once the drier had been preheated to the specified temperature (140, 160, or 180 °C), two Petri dishes with rapeseeds from each variant were placed in the drier and were heated for the specified time (5, 10, or 15 min). Immediately after their removal from the drier, the seeds were transferred to a cold glass container. After the seeds had cooled to ambient temperature (approximately 18 °C), all samples with the same temperature and roasting duration were combined and mixed. The water content of the roasted seeds was measured and the samples were moisturized up to 6%. The quantity of added water was measured using a material balance. The samples were stored in closed polyethylene bags for 24 h. After that time, oil was pressed from the whole seeds. Rapeseed was pressed at room temperature using a Farmet Uno cold-pressing machine (Farmet, Czech Republic). The temperature inside the press was 60 ± 10 °C and the temperature of the produced oil was 39 ± 1 °C. The oil was centrifuged at 5000 rpm for 15 min and transferred directly to small dark 100 ml bottles and stored at 4 °C in the dark.

### Peroxide Value (PV) and Acid Value (AV) Determination

The PV and AV of the cold-pressed rapeseed oils were determined using the standard methods from ISO 3960:2001 (Animal and vegetable fats and oils: determination of peroxide value) [33] and ISO 660:1996 (Animal and vegetable fats and oils: determination of acid value and acidity) [34].

### Determination of Tocopherols, Plastochromanol-8, and Canolol by NP-HPLC

Rapeseed oil (200 mg) was dissolved in *n*-hexane, made up to 10 ml, and transferred to vials for analysis. Tocopherols were qualitatively and quantitatively identified using a Waters HPLC system (Waters, Milford, MA) consisting of a pump (Waters 600), a fluorimetric detector (Waters 474), a photodiode array detector (Waters 2998 PDA), an autosampler (Waters 2707), a column oven (Waters Jetstream 2 Plus), and a LiChrosorb Si 60 column (250 × 4.6 mm, 5 µm) from Merck (Darmstadt, Germany). The mobile phase was a mixture of *n*-hexane with 1.4-dioxane (96:4 *v/v*). The flow rates were 1.0 ml/min (for tocopherols and PC-8) and 2.0 ml/min (for canolol). To detect the fluorescence of tocopherols and PC-8, the excitation wavelength was set at λ = 295 nm and the emission wavelength at λ = 330 nm. To detect the fluorescence of canolol, the excitation wavelength was set at λ = 280 nm and the emission wavelength at λ = 325 nm. Standards of α-, β-, γ- and δ-tocopherols (>95% of purity) were purchased from Merck (Darmstadt, Germany). The plastochromanol-8 (PC8) contents were assayed and calculated following to Siger *et al*. [35].

### Statistical Analysis

The experiment with the roasting rape seeds was performed twice. Results are presented as means ± standard deviations from three replicates for each bioactive compound analysis. The differences between the mean values were determined by analysis of variance (ANOVA). The *post hoc* analysis was performed using Tukey’s test. Intra-sample quantity variation of the oils was assayed using principal component analysis (PCA). The results of all tests were considered significant at *p* < 0.05. The statistical analysis was performed using Statistica 10.0 software (StatSoft, Inc., Tulsa, OK).

## Results and Discussion

The protein contained in the pomace left behind after pressing the rapeseed oil could receive better and broader use if the seeds had a lower fiber content, as fiber reduces the energy value of the pressed oil. The solution to this problem is to breed yellow-seeded varieties, which have thinner seed coats and lower concentrations of polyphenolic compounds, with higher contents of protein and fat. Such features are very desirable and reflect the effectively of breeding aimed at improved the quality characteristics of rape [36, 37].

The seeds' fat content was determined as 46.7 ± 0.51% in the case of the line PN1 03/1i/14 and 46.6 ± 0.35% for the line PN1 563/1i/14. The seeds of line PN1 03/1i/14 were found to contain 20.8 ± 0.41% protein, and the seeds of line PN1 563/1i/14 had 21.4 ± 0.36% protein. Neutral detergent fiber (NDF) and acid detergent fiber (ADF) were also analyzed. In the line PN1 03/1i/14, the levels of NDF and ADF were lower than in the line PN1 563/1i/14 (19.04 ± 0.21% as against 20.41 ± 0.26 and 12.14 ± 0.31% against 14.05 ± 0.14%, respectively). The total glucosinolate content was 15.25 ± 0.17 M/g free fatty dry mater (ffdm) of the seeds (including 6.61 ± 0.21 M/g ffdm alkenyl glucosinolates) for the seeds of line PN1 03/1i/14 and 15.31 ± 0.15 M/g ffdm (including 9.39 ± 0.17 M/g ffdm alkenyl glucosinolates) for the seeds of line PN1 563/1i/14 (data not show).

### Changes in Acid Value and Peroxide Value

According to the Codex Alimentarius [38], cold-pressed rapeseed oil should have an acid value (AV) < 4 mg KOH/kg, while its peroxide value should be LOO < 15 meq O_2_/kg. The peroxide values for the cold-pressed oil from the unroasted seeds (the control sample) were 1.27 and 1.20 meq O_2_/kg, for the seeds of line PN1 03/1i/14 and seeds of line PN1 563/1i/14, respectively. Roasting the seeds resulted in an increase in the peroxide content of the oils obtained from both lines of yellow-seeded rapeseed. Roasting the seeds at 140 °C caused a statistically significant increase (*p* < 0.0001) in peroxide over that of the control sample. The peroxide levels increased to 2.31 meq O_2_/kg in the oil from the line PN1 03/1i/14 and to 1.83 meq O_2_/kg in that from the line PN1 563/1i/14. Increasing the roasting temperature to 160 °C caused further a statistically significant (*p* < 0.0001) increase in the peroxide levels of the oils. In the case of the seeds line PN1 03/1i/14, following 15 min of roasting at 160 °C, the peroxide content reached 3.09 meq O_2_/kg. For the seeds line PN1 563/1i/14, peroxide reached 2.55 meq O_2_/kg (Table 1). Increasing the temperature to 180 °C did not cause a significant difference (*p* > 0.05) in peroxide content over that of the seeds roasted at 160 °C. It should be noted that the peroxide content of the oil from the line PN1 563/1i/14—which have a darker seed coat—is lower than that from the line PN1 03/1i/14, which have brighter seed coats. The free fatty acid contents show no substantial difference (*p* > 0.05) between roasted seeds and unroasted seeds (Table 1). The acid values for the control samples were 1.15 and 1.30 mg KOH/g for cold-pressed oil from the seeds of line PN1 03/1i/14 and seeds of line PN1 563/1i/14, respectively. Roasting the seeds caused a small increase in the acid value. In the case of the oil from the roasted seeds of line PN1 03/1i/14, the acid values ranged between 1.21 and 1.47 mg KOH/g. In contrast, the line PN1 563/1i/14 seeds acid value varied from 1.27 to 1.41 mg KOH/g (Table 1). According to Ghazani *et al*. [39], cold-pressed canola oils had lower free fatty acid concentrations, peroxide values, p-anisidine values, and chlorophyll levels than solvent-extracted and hot-pressed canola oils. In earlier research, Siger *et al*. [30] analyzed cold-pressed rapeseed oil obtained from black-seeded rapeseed. The pressing and roasting of the seeds was performed under the same conditions. That study did not show any statistically significant differences in peroxide content or free fatty acid content between the oil from the control seeds and the oils from roasted seeds. For the oils from black-seeded rapeseed, the peroxide values were higher, in the 2.03–3.48 meq O_2_/kg range. On the other hand, the acid values for black-seeded rapeseed oil were lower and ranged from 0.51 to 0.68 mg KOH/g. Gawrysiak-Witulska *et al*. [15], by researching the effect of drying yellow-seeded rapeseeds, showed in a control sample (of oil from undried seeds) that the free fatty acid content was 1.13 mg KOH/g. Drying at 40 and 60 °C did not result in any statistically significant changes in the free fatty acid content. On the other hand, drying at 80–120 °C caused an increase in the acid value from 1.48 to 1.68 mg KOH/g.

**Table 1:** Peroxide values and acid values of cold-pressed rapeseed oils from roasted yellow-seeded *Brassica napus*

Oil source	Peroxide value (meq O_2_/kg)		Acid value (mg KOH/g)	
	Line PN1 03/1i/14	Line PN1 563/1i/14	Line PN1 03/1i/14	Line PN1 563/1i/14
Unroasted seeds (control)	1.27 ± 0.23^a^	1.20 ± 0.13^a^	1.15 ± 0.12^a^	1.30 ± 0.15^a^
Seeds roasted at 140 °C/5 min	1.83 ± 0.18^b^	1.27 ± 0.14^a^	1.37 ± 0.14^a^	1.27 ± 0.10^a^
Seeds roasted at 140 °C/10 min	2.31 ± 0.20^c,d^	1.83 ± 0.21^c^	1.36 ± 0.16^a^	1.30 ± 0.11^a^
Seeds roasted at 140 °C/15 min	2.07 ± 0.15^b,c^	1.64 ± 0.13^b^	1.35 ± 0.17^a^	1.27 ± 0.17^a^
Seeds roasted at 160 °C/5 min	2.22 ± 0.24^c,d^	1.97 ± 0.27^c,d^	1.21 ± 0.11^a^	1.37 ± 0.14^a^
Seeds roasted at 160 °C/10 min	3.07 ± 0.16^f^	2.51 ± 0.15^f^	1.30 ± 0.15^a^	1.40 ± 0.15^a^
Seeds roasted at 160 °C/15 min	3.09 ± 0.17^f^	2.55 ± 0.16^f^	1.41 ± 0.12^a^	1.41 ± 0.12^a^
Seeds roasted at 180 °C/5 min	2.69 ± 0.14^d,e^	1.93 ± 0.17^c,d^	1.37 ± 0.14^a^	1.35 ± 0.11^a^
Seeds roasted at 180 °C/10 min	2.82 ± 0.13^e,f^	2.15 ± 0.16^d,e^	1.36 ± 0.13^a^	1.37 ± 0.11^a^
Seeds roasted at 180 °C/15 min	2.80 ± 0.18^e,f^	2.33 ± 0.10^e,f^	1.47 ± 0.14^a^	1.38 ± 0.16^a^

Values (means ± SD) bearing different superscripts are statistically significantly different (*p* < 0.05)

### Changes in Canolol Content

There are data in the literature on the impact of the roasting process on the rate of decarboxylation of sinapic acid in seeds of rape, though only for the black-seeded variety. The difference with yellow seeds arises from their much thinner seed coats—the result of a reduction in fiber content, which in turn makes the coat transparent and causes the yellow embryo to become visible. Both the seed color and the indigestible fiber content are associated with the biochemistry of phenylpropanoids, which are the precursors to or components of phenolic compounds [10]. For this reason, it would also be interesting to determine how seeds with a thinner seed coat would react to high temperatures (including the formation of canolol) [26–30].

The changes in the canolol content are shown in Table 2. In the cold-pressed oils from the unroasted seeds, the canolol concentration was 8.53 and 12.45 μg/g of oil, respectively, for oil from seeds of line PN1 03/1i/14 and from seeds of line PN1 563/1i/14. The canolol contents of black-seeded control samples depended on the experiment: 5.8 μg/g [26], 24.74 μg/g [28], 5.19 μg/g [27], 17.06 μg/g [29], or 11.54 μg/g [30]. Roasting the seeds caused a statistically significant increase (*p* < 0.0001) in the canolol content of the oil. The canolol content was influenced both by the increase in temperature and in the roasting time. In the case of the line PN1 03/1i/14, roasting at 140 °C led to an increase in canolol to 22.36 μg/g after 5 min of roasting, to 45.44 μg/g after 10 min of roasting, and to 132.28 μg/g after 15 min of roasting. Increasing the temperature to 160 °C caused a further increase in the canolol content of the oil. After 15 min of roasting, the canolol content increased to 258.93 μg/g (30 times that obtained from the control sample). In oil obtained from seeds roasted at 180 °C, the canolol contents after 5 and 10 min were 264.32 μg/g and 473.65 μg/g, respectively. The highest canolol levels of 768.26 μg/g were noted after roasting the seeds of the line PN1 03/1i/14 for 15 min; this is 90 times the value from the control sample. A smaller increase in canolol was noted in the oil pressed from the roasted seeds of line PN1 563/1i/14: a eightfold increase in canolol was seen for the oil from line PN1 563/1i/14 roasted at 140 °C for 15 min. After 5 min of roasting at 160 °C, the canolol content in the oil was 58.72 μg/g; after 10 min, 137.42 μg/g; and after 15 min, 222.65 μg/g (Table 2). The canolol content in the oil from the seeds roasted at 180 °C for 5 min was 229.69 μg/g. Increasing the roasting time to 10 min resulted in a canolol content of 394.74 μg/g; after 15 min, 576.43 μg/g was obtained (46 times more than in the control sample). This is 192 μg/g less than in the case of the seeds of line PN1 03/1i/14. A detailed analysis of the results shows that, over the range of the applied roasting temperatures and times, a given canolol value can be achieved by increasing the roasting temperature or by lengthening the time of exposure at a lower temperature. For example, in the case of seeds roasted at 160 °C for 10 min, the canolol value was similar to that of seeds roasted at 140 °C for 15 min. A similar situation occurs in the case of the oils from seeds roasted at 160 °C for 15 min as compared with those roasted for 180 °C for 5 min (Table 2). This was not observed in the case of the black-seed oils during previous studies [30]. The difference in canolol content between yellow seeds and brown seeds may be the result of their different NDF, ADF, and alkenyl glucosinolate contents, which are higher in brown seeds. According to the study of Spielmeyer *et al*. [26], the highest amount of canolol can be found in the oil from seeds roasted at 160 °C; above this temperature, the canolol content falls. However, studies of other compounds fail confirm this dependence. Shrestha and De Meulenaer [27] roasted rapeseeds at 180 °C for 10–90 min and noted the highest canolol value at 20 min. Shrestha *et al*. [28], after 10 min of roasting rapeseeds at 180 °C, showed 707.69 μg/g canolol extracted with organic solvents (petroleum ether); they also obtained 790.41 μg/g canolol from roasted (flaked) seeds. Research on black rapeseed roasted under exactly the same conditions showed canolol contents of 84.58 μg/g at 140 °C and 244.76 μg/g at 160 °C. At 180 °C, they also noted an increase in canolol levels from 328.98 to 609.94 μg/g [30]. Comparing the canolol content of the oil from the yellow-colored seeds of line PN1 03/1i/14 with the results of the earlier study on the oil from black-colored seeds, it can be seen that, when roasted for only 5 min, independent of the temperature, the oil from the yellow-colored seeds has less canolol than that from the black-colored seeds (results shown in a previous study [30]). When the roasting time is extended to 15 min, the oil from the seeds of line PN1 03/1i/14 has a higher canolol content than that from the black seeds. This can be explained by the fact that the line PN1 03/1i/14 (yellow seeds) have less phenolic compounds and have a thinner seed coat. According to Rahman and Joersbo [40], the seed coat consists of several layers: the epidermal layer is covered by the epithelium, under which lie the compressed subepidermal layers, the palisade cell layers, the compact pigment layers, and a single aleurone layer. The pigments are mainly contained in the palisade and pigment layers which, together with the clear, thin epidermis, are derived from the integument of the ovule, and thus from the ovule’s maternal tissue. A significant amount of fiber is concentrated in the palisade layers, which in the case of the yellow seeds are reduced by about half to two-thirds of this natural thickness, which reduces the polyphenol and lignin content by a similar degree. Li *et al*. [41] also reported that the yellow-seeded lines they used, compared to traditional rapeseed cultivars, showed lower total levels of cellulose, phenylalanine, and tyrosine, as well as of pigments (chlorophyll, carotenoids, and melanin). There were also changes in the levels of secondary metabolites, such as coumarin, caffeine, and others, and in the activity of enzymes such as polyphenol oxidase, phenylalanine ammonia-lyase, tyrosinase, peroxidase, and a number of dehydrogenases, hydrolases, and ligases related to the synthesis of the various pigments. The synthesis of pigments in plants happens mainly through the shikimate pathway, key roles in which are played by phenylalanine ammonia-lyase, polyphenol oxidase and peroxidase. The first of these carries out the deamination of phenylalanine to phenylacrylic acid, a polyphenol precursor [42].

**Table 2:** Canolol contents of cold-pressed oils from unroasted seeds and seeds roasted yellow-seeded *Brassica napus* at different temperature and time variants

Oil source	Canolol content (μg/g)			
	Line PN1 03/1i/14	(%)	Line PN1 563/1i/14	(%)
Unroasted seeds (control)	8.53 ± 0.65^a^	100	12.45 ± 0.59^a^	100
Seeds roasted at 140 °C/5 min	22.36 ± 0.36^b^	262	39.28 ± 0.78^b^	316
Seeds roasted at 140 °C/10 min	45.44 ± 0.85^c^	533	60.73 ± 0.41^d^	488
Seeds roasted at 140 °C/15 min	132.28 ± 0.65^e^	1551	97.33 ± 0.96^e^	782
Seeds roasted at 160 °C/5 min	64.73 ± 0.47^d^	759	58.72 ± 1.02^c^	472
Seeds roasted at 160 °C/10 min	147.23 ± 0.85^f^	1726	137.42 ± 1.24^f^	1104
Seeds roasted at 160 °C/15 min	258.93 ± 1.69^g^	3036	222.65 ± 1.95^g^	1788
Seeds roasted at 180 °C/5 min	264.32 ± 1.75^h^	3075	229.69 ± 0.98^h^	1813
Seeds roasted at 180 °C/10 min	473.65 ± 1.45^i^	5553	394.74 ± 2.41^i^	3171
Seeds roasted at 180 °C/15 min	768.26 ± 2.58^j^	9007	576.43 ± 2.74^j^	4630

Values (means ± SD) bearing different superscripts are statistically significantly different (*p* < 0.05)

### Tocochromanol Content

When investigating the effect of temperature on the rate of decarboxylation of sinapic acid in yellow-seeded lines of rape, it is necessary at the same time to study the effect of the modification used in the preparing the seeds—namely, of roasting—on the other biologically active compounds present in rape, such as tocochromanols.

The results are shown in Tables 3 and 4. The oil obtained from the unroasted seeds of line PN1 03/1i/14 was characterized by higher tocopherol and plastochromanol-8 values than that obtained from the unroasted seeds of line PN1 563/1i/14. Briefly, the tocopherol content in the control sample was 73.23 mg/100 g of oil from the line PN1 03/1i/14 and 65.58 mg/100 g of oil from the line PN1 563/1i/14 (Tables 3, 4). In both lines, the dominant homologues were γ-T and α-T. In the case of the line PN1 03/1i/14, the γ-T content was 39.27 mg/100 g, and the level of α-T was 33.07 mg/100 g. Oil from the seeds line PN1 563/1i/14 was characterized by a γ-T content of 35.67 mg/100 g and an α-T content of 29.32 mg/100 g. The rest of the homologues occurred in amounts not exceeding 1 mg/100 g of oil. The plastochromanol-8 content of the oil from the line PN1 03/1i/14 was higher than that from the line PN1 563/1i/14, amounting to 2.53 mg/100 g. In the line PN1 563/1i/14-seed oil, the level of PC-8 did not exceed 2 mg/100 g. The β-T and δ-T contents of the roasted yellow-seeded rapeseed were not statistically significant (*p* > 0.05). In the roasting at 140 °C for 5 or 10 min, the α-T and γ-T homologue contents were statistically significantly lower (*p* < 0.0001) than in the control sample. The content of γ-T in the oil from seeds (line PN1 03/1i/14) roasted at 140 °C for 15 min was statistically significantly higher (*p* < 0.0001) than in the case of the oils from the seeds roasted at the same temperature but for only 5 or 10 min (Table 3). Increasing the roasting temperature to 160 °C did not lead to any substantial differences in the γ-T homologue content of the line PN1 03/1i/14 -seed oil. However, the oils obtained from the seeds (line PN1 03/1i/14) roasted at 180 °C for 10 or 15 min had statistically significant differences in their γ-T homologue content. This increased by 7% in the case of 10 min roasting and by 17% in the case of 15 min roasting (Table 3). As a result, it can be seen that the oil samples taken from seeds roasted at 180 °C for 10 and 15 min contain statistically significantly more (*p* < 0.0001) of these compounds, at 76.46 and 81.34 mg/100 g, respectively.

**Table 3:** Tocopherol contents of cold-pressed oils from roasted yellow-seeded *Brassica napus* (line PN1 03/1i/14)

Oils	Tocopherol contents (mg/100 g)					PC-8 content (mg/100 g)
	α-T	β-T	γ-T	δ-T	Total	
Unroasted seeds (control)	33.07 ± 0.23^c^	0.11 ± 0.01^a^	39.27 ± 0.14^c^	0.78 ± 0.09^a,b^	73.23 ± 0.27^c^	2.53 ± 0.14^a^
Seeds roasted at 140 °C/5 min	31.94 ± 0.21^a^	0.11 ± 0.03^a^	37.17 ± 0.23^a^	0.70 ± 0.11^a^	69.92 ± 0.26^a^	2.65 ± 0.11^a^
Seeds roasted at 140 °C/10 min	31.72 ± 0.15^a^	0.07 ± 0.04^a^	37.02 ± 0.32^a^	0.73 ± 0.08^a^	69.54 ± 0.32^a^	2.72 ± 0.12^a^
Seeds roasted at 140 °C/15 min	32.47 ± 0.14^b^	0.11 ± 0.02^a^	38.93 ± 0.14^b^	0.74 ± 0.14^a,b^	72.25 ± 0.47^b,c^	3.02 ± 0.25^a,b^
Seeds roasted at 160 °C/5 min	32.07 ± 0.21^b^	0.07 ± 0.03^a^	37.43 ± 0.16^a^	0.68 ± 0.01^a^	70.25 ± 0.26^a^	3.19 ± 0.18^b,c^
Seeds roasted at 160 °C/10 min	32.43 ± 0.11^b^	0.07 ± 0.01^a^	39.01 ± 0.21^c^	0.76 ± 0.06^a,b^	72.27 ± 0.34^b,c^	3.68 ± 0.11^c,d^
Seeds roasted at 160 °C/15 min	32.13 ± 0.20^b^	0.04 ± 0.06^a^	39.23 ± 0.19^c^	0.70 ± 0.10^a,b^	72.10 ± 0.26^b^	3.86 ± 0.17^d^
Seeds roasted at 180 °C/5 min	32.18 ± 0.19^b^	0.07 ± 0.02^a^	40.17 ± 0.11^d^	0.69 ± 0.08^a,b^	73.11 ± 0.32^c^	4.57 ± 0.22^e^
Seeds roasted at 180 °C/10 min	33.43 ± 0.23^c^	0.09 ± 0.05^a^	42.14 ± 0.19^e^	0.80 ± 0.11^b^	76.46 ± 0.37^d^	4.86 ± 0.16^e,f^
Seeds roasted at 180 °C/15 min	33.47 ± 0.22^c^	0.07 ± 0.08^a^	46.17 ± 0.26^f^	0.93 ± 0.09^b^	81.34 ± 0.29^e^	5.63 ± 0.22^f^

Values (means ± SD) bearing different superscripts are statistically significantly different (*p* < 0.05)

**Table 4:** Tocopherol contents of cold-pressed oils from roasted yellow-seeded *Brassica napus* (line PN1 563/1i/14)

Oils	Tocopherol contents (mg/100 g)					PC-8 content (mg/100 g)
	α-T	β-T	γ-T	δ-T	Total	
Unroasted seeds (control)	29.32 ± 0.17^a^	0.08 ± 0.07^a^	35.67 ± 0.21^a^	0.51 ± 0.09^a^	65.58 ± 0.25^b^	21.98 ± 0.12^a^
Seeds roasted at 140 °C/5 min	29.23 ± 0.19^a^	0.08 ± 0.01^a^	35.73 ± 0.22^a^	0.49 ± 0.05^a^	65.53 ± 0.32^b^	2.18 ± 0.13^a,b^
Seeds roasted at 140 °C/10 min	29.13 ± 0.16^a^	0.05 ± 0.02^a^	35.20 ± 0.14^a^	0.47 ± 0.10^a^	64.85 ± 0.25^a^	2.22 ± 0.10^a,b^
Seeds roasted at 140 °C/15 min	29.36 ± 0.21^a,b^	0.65 ± 0.02^a^	35.99 ± 0.26^a,b^	0.40 ± 0.11^a^	65.72 ± 0.22^b^	2.20 ± 0.20^a,b^
Seeds roasted at 160 °C/5 min	29.96 ± 0.27^b,c^	0.03 ± 0.01^a^	36.13 ± 0.13^b^	0.41 ± 0.08^a^	66.53 ± 0.34^c^	2.57 ± 0.12^b^
Seeds roasted at 160 °C/10 min	29.76 ± 0.22^b,c^	0.04 ± 0.01^a^	36.17 ± 0.14^a,b^	0.42 ± 0.07^a^	66.20 ± 0.28^c^	3.19 ± 0.20^c^
Seeds roasted at 160 °C/15 min	29.86 ± 0.16^b,c^	0.03 ± 0.01^a^	36.26 ± 0.25^b^	0.45 ± 0.10^a^	66.60 ± 0.26^c^	3.29 ± 0.17^c^
Seeds roasted at 180 °C/5 min	30.17 ± 0.16^c^	0.05 ± 0.03^a^	37.53 ± 0.19^c^	0.43 ± 0.08^a^	68.18 ± 0.36^d^	3.48 ± 0.16^c^
Seeds roasted at 180 °C/10 min	32.88 ± 0.11^d^	0.04 ± 0.02^a^	39.28 ± 0.16^d^	0.47 ± 0.11^a^	72.67 ± 0.34^e^	3.96 ± 0.20^d^
Seeds roasted at 180 °C/15 min	32.76 ± 0.25^d^	0.06 ± 0.05^a^	42.17 ± 0.17^e^	0.48 ± 0.12^a^	75.47 ± 0.25^f^	4.21 ± 0.18^d^

Values (means ± SD) bearing different superscripts are statistically significantly different (P < 0.05)

It can be seen that the α-T content of the oils from line PN1 563/1i/14 seeds roasted at 140 °C for 5 and 10 min does not statistically significantly differ (*p* > 0.05) from the control sample. However, in the other samples, an increase in α-T can be seen; this was not noted in the line PN1 03/1i/14-seed oils. In the case of the oils from the line PN1 563/1i/14 seeds that had been roasted at 180 °C for 10 and 15 min, a 12% increase in the α-T content was noted (Table 4). It was shown that the content of the γ-T homologue increased with temperature and roasting time. No statistically significant difference was noted (*p* > 0.05) between the oils from seeds roasted at 140 °C and the control sample. Roasting at 160 °C led to a statistically significant difference (*p* < 0.0001) in the levels of this homologue, which exceeded 36 mg/100 g (Table 4). The greatest increase in this homologue was obtained for seeds roasted at 180 °C for 10 or 15 min, similarly to the seeds of line PN1 03/1i/14. In the oil from roasted seeds, a 10% increase in γ-T was noted after 10 min and an 18% increase after 15 min.

The two lines of yellow rapeseed studied here also differed in their PC-8 content. The oil from the seeds of line PN1 03/1i/14 contained between 2.53 and 5.63 mg/100 g of this compound, and that from seeds of line PN1 563/1i/14 had 1.98–4.21 mg/100 g (Tables 3, 4). In both cases, it was shown that the PC-8 content of the oils from roasted seeds increased with both the temperature and the time. An increase in PC-8 was noted for the oil from both lines of seeds when roasted at 140 °C, though this was not statistically significant. Increasing the temperature to 160 °C led to a further increase in the amount of this compound, the higher the more the temperature and time increased. The PC-8 content in the oils from the line PN1 03/1i/14 seeds roasted at 160 °C ranged from 2.65 (at 5 min roasting) to 3.02 mg/100 g (at 15 min roasting). In the oils from the seeds roasted at 180 °C for 15 min, the PC-8 content increased to 5.63 mg/100 g. A similar situation was observed with the seeds of line PN1 563/1i/14 which, when roasted at 160 °C yielded a statistically significant increase (*p* < 0.0001) in PC-8: 29% greater after 5 min roasting, 61% after 10 min roasting, and 66% after 15 min roasting. In the oils from the seeds roasted at 180 °C for 10 and 15 min, a 100% increase in PC-8 was seen, reaching 4.21 mg/100 g. No statistically significant difference (*p* > 0.05) was shown among the oils from the seeds roasted at 180 °C for 10 and 15 min. Gawrysiak-Witulska *et al*. [15] studied the changes in tocochromanol in yellow rapeseed during the phases of postharvest processing, taking into account the drying of seeds under different temperature conditions (40, 60, 80, 100, and 120 °C). They showed that the initial tocopherol content of the seeds after collection was 610.5 mg/kg of oil. In seeds subsequently dried with air at 40 and 60 °C, the total losses of tocopherols were less than 1% and statistically negligible. Increasing the drying temperature to 80 °C increased the tocopherol loss to 13%, and heating to 100 and 120 °C caused the tocopherol losses to increase to 23%. During drying at 40 and 60 °C, the losses of α-T, β-T, and γ-T did not exceed 2% and the loss of δ-T was at 6%. Increasing the drying air temperature to 80 °C increased the losses of the individual tocopherols above 10%. With drying at 100 and 120 °C, the losses of α-T and γ-T (the major tocopherols in rapeseed) were similar, at 23% [15]. In the case of the oils made from the yellow seeds roasted at 140 °C for 5 and 10 min, there was a loss of around 4%. At higher temperatures and for longer roasting, an increase in these compounds was observed. Shrestha and Meulenaer [27] analyzed the tocochromanol content of oil extracted from mustard seeds and rapeseed that had been subjected to the roasting process in oil baths at 180 °C prior to extraction with solvent. The oil from rapeseeds that had not been roasted contained 214.89 μg/g α-T and 274.14 μg/g γ-T, while the oil from the roasted seeds, independent of the roasting length, contained around 10% more of the α-T homologue. In the case of the γ-T homologue, the authors saw a 6–7% decrease. For mustard seeds, they showed an 85% decrease in α-T, which was proportional to the roasting time. For the γ-T homologue, this decrease was reached a maximum at around 40%. By analyzing the PC-8 content of oil extracted from rapeseeds, Shrestha and Meulenaer [27] showed an increase in the compound from 1.8 μg/g of oil to 48.01 μg/g of oil from seeds roasted for 45 min. These authors explain the increase in the tocochromanol content in these oils by suggesting that, during roasting, the matrix is disturbed, which results in better extraction of certain tocochromanols. Wijesundera *et al*. [43] investigated the effect on the tocochromanol content of roasting black-seeded rapeseeds and mustard seeds at 165 °C for 5 min. They concluded that there was no effect of roasting on the content of the α-T homologue, but that the level of γ-T showed a value higher by around 10% for the strains of rapeseed and mustard they tested. This apparent increase is possibly due to its coelution in HPLC with another component generated after roasting. Recent work has shown that the tocopherol content of oils extracted from “light” and “medium” roasted seed degraded more rapidly than that of oil from raw seeds. Neither Wakamatsu *et al*. [25] nor Spielmeyer *et al*. [26] found any significant differences between the tocopherol content of canola oil from unroasted and roasted raw material. Matthäus [44] suggested that the canolol formed during roasting may protect tocopherols against degradation at elevated temperatures. Roasting of different rapeseed varieties (a typical high linolenic rapeseed variety “Brandy” and “PR46W20”; high oleic acid rapeseed HO PN1414;) has shown that the content of γ-T and PC-8 is always highest when the roasting is at temperature 180°C. At the same time the content of canolol is the highest. It seems that there is a synergistic relationship between the canolol content and the amount of tocopherols [30, 45]. The increase in oxidative stability and antioxidant activity is probably also influenced by Maillard reaction products. Maillard reaction products with antioxidant properties generated during roasting may also contribute to the higher stability of oil to oxidation [27, 46].

**Fig. 1:**
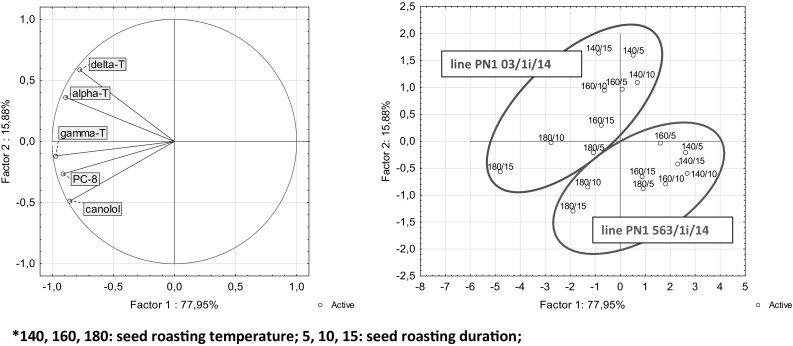
Two first principal components after PCA analysis of alpha-, gamma-, delta-tocopherol, plastochromanol-8 and canolol contents of cold-pressed oils from roasted yellow-seeded *Brassica napus*. 140, 160, 180 C: seed roasting temperature; 5, 10, 15 min: seed roasting duration. The lines used in the experiments were characterized by different seed colors: line PN1 03/1i/14 was *yellow* and line PN1 563/1i/14 was *brown*

The statistical analysis has confirmed the significant influence of temperature and time of roasting on particular bioactive component levels in sampled oils. It was found that with the increases in both temperature and roasting time cause the increase in canolol, tocopherols and plastochromanol-8 levels. It was found the significant correlation between canolol and tocopherols and PC-8. For line PN1 03/1i/14, a statistically significant correlation was observed between canolol content and α-T levels (*r* = 0.6715; *p* = 0.0035), γ-T levels (*r* = 0.9755; *p* < 0.0001), total tocopherols (*r* = 0.9464; *p* < 0.0001) and PC-8 (*r* = 0.9182; *p* < 0.0001). The results recorded for line PN1 563/1i/14 show that canolol levels are correlated with α-T levels (*r* = 0.8227; *p* = 0.0068), γ-T levels (*r* = 0.9321; *p* < 0.0001), total tocopherols (*r* = 0.8996; *p* < 0.0001), and PC-8 (*r* = 0.8912; *p* < 0.0001). To show the differences between new breeding line yellow-seeded *Brassica napus* a principal component analysis was performed. Principal component analysis confirmed the variation in the tested oils obtained from two new breeding lines of yellow-seeded rapeseed, differing by seed-coat color: line PN1 03/1i/14 (yellow) and line PN1 563/1i/14 (brown) (Fig. 1). The greatest effect on factor 1 in PCA is negatively correlated with γ-T and α-T levels (−0.97; −0.89, respectively), PC-8 (−0.91) and canolol content (−0.89). In turn, factor 2 is correlated to the greatest degree with δ-T content (0.58) and negatively correlated with canolol content (−0.48). Principal component analysis confirmed the differences in the content of native antioxidants in oils obtained from yellow-seeded rapeseed, differing by seed-coat color. The oil obtained from the seeds of the line PN1 03/1i/14 (yellow seeds) has a higher increase in canolol during the roasting process. The content of bioactive components especially canolol depends on the thickness of the hull. Line PN1 03/1i/14 seeds have a thinner hull than the normal brown or black seed coat of the brassicas, the fiber content is reduced and an oil and protein rich embryo makes up a larger proportion of the seed. Thinner hulls cause better heat absorption what favors the decarboxylation sinapic acid process. The increase in γ-T and PC-8 needs further investigation. It is worth knowinf that if the matrix destruction caused by roasting caused better extraction of certain tocochromanols or this increase of γ-T and PC-8 is caused by coelution in HPLC with another component generated after roasting.

## Conclusions

Our study has shown that oil pressed from roasted yellow rapeseeds is of good quality, as confirmed by its low peroxide and acid values. It was concluded that, the higher the roasting temperature, the higher the canolol content of the oil. However, this increase was different for seeds with different seed-case colors. In oil obtained from seeds of line PN1 03/1i/14, the amount of this compound increased 90-fold in relation to the control sample. In the case of seeds of line PN1 563/1i/14, the increase in canolol was no more than 46 times greater. Changes in tocochromanol contents were observed between the studied lines of yellow rapeseed. In the oils obtained from the seeds roasted at 180 °C for 10 and 15 min, increases were observed especially in the γ-T homologue and in plastochromanol-8. Comparing rapeseed lines of different colors, it can be concluded that roasting the line PN1 03/1i/14 rapeseed prior to the cold pressing process allows oil with a higher content of bioactive compounds (mainly canolol) to be obtained than in the case of darker-hulled rapeseed (line PN1 563/1i/14).

## Abbreviations

PV: Peroxide value
AV: Acid value
PC-8: Plastochromanol-8
T: Tocopherol
α-T: Alpha-tocopherol
β-T: Beta-tocopherol
γ-T: Gamma-tocopherol
δ-T: Delta-tocopherol
